# Statement of retraction: Tumor-associated fibroblasts derived exosomes induce the proliferation and cisplatin resistance in esophageal squamous cell carcinoma cells through RIG-I/IFN-β signaling

**DOI:** 10.1080/21655979.2026.2731275

**Published:** 2026-10-01

We, the Editors and Publisher of the journal *Bioengineered*, have retracted the following article from publication.

Cui, Y., Zhang, S., Hu, X., & Gao, F. (2022). Tumor-associated fibroblasts derived exosomes induce the proliferation and cisplatin resistance in esophageal squamous cell carcinoma cells through RIG-I/IFN-β signaling. *Bioengineered, 13*(5), 12462–12474. https://doi.org/10.1080/21655979.2022.2076008

Since publication, significant concerns have been raised by a third party about the integrity of the data and reported results in the article.

When approached for an explanation, the authors have not responded to our queries and so these serious concerns remain unaddressed.

As verifying the validity of published work is core to the integrity of the scholarly record, we are therefore retracting the article. The authors have been informed of this decision.

We have been informed in our decision-making by our policy on publishing ethics and integrity and COPE guidelines. 

The retracted article will remain online to maintain the scholarly record and will be digitally watermarked on each page as ‘Retracted’. 

